# Development of a fully automated high throughput PCR for the detection of SARS-CoV-2: The need for speed

**DOI:** 10.1080/21505594.2020.1798041

**Published:** 2020-07-29

**Authors:** Florian J. Mayer, Franz Ratzinger, Ralf L.J. Schmidt, Georg Greiner, Olfert Landt, Alexander Am Ende, Victor M. Corman, Nicole Perkmann-Nagele, Thomas Watkins-Riedel, Dagmar Petermann, Karoline Abadir, Josef Zweimüller-Mayer, Robert Strassl

**Affiliations:** a Divison of Clinical Virology, Department of Laboratory Medicine, Medical University of Vienna, Vienna, Austria; b Ihr Labor, Medical Diagnostic Laboratories, Vienna, Austria; c Tib-Molbiol, Berlin, Germany; d Charité – Universitätsmedizin Berlin Institute of Virology, Berlin, Germany and German Centre for Infection Research (DZIF), Berlin, Germany; e QIAGEN GmbH, Vienna, Austria

**Keywords:** COVID-19, SARS-CoV-2, Coronavirus

## Abstract

Currently, testing for coronavirus is performed with time and personnel consuming PCR assays. The aim of this study was to evaluate the sensitivity, specificity and capacity of a fully automated, random access high-throughput real-time PCR-based diagnostic platform for the detection of SARS-CoV-2. The *NeuMoDx N96* system displayed an equal or better detection rate for SARS-CoV-2 compared with the *LightCycler 480II* system and showed a specificity of 100%. The median PCR run time for all 28 PCR runs was 91 (IQR 84–97) minutes. The capacity of the *NeuMoDx N96* could easily surpass the capacity of most currently used molecular test systems and significantly reduce the turn-around time.

## Introduction

Several research groups undertook a tremendous amount of effort to identify the sequence of the newly emerged pandemic severe acute respiratory syndrome coronavirus 2 (SARS-CoV-2). Just a few days after the viral genome was available [1], first laboratory-validated real-time PCR assays were published targeting E gene, N gene and RdRP [2] and the orf1b and N gene [3].

Currently, testing for coronavirus is performed with time and personal consuming in-house PCR assays which typically need a lot of manual steps and require about 3 to 4 hours to the final test result. Reusken et al. (published in January 2020) assessed the diagnostic capacity of specialized laboratories of 30 European countries and the results are more than alarming: 46 laboratories had a combined capacity of only 9,150 tests per week [4]. Considering the current ongoing spread in Europe, especially in Italy, an outbreak of Coronavirus with similar or higher rates of infections as in China is not inconceivable. Under these circumstances, laboratories across Europe will rapidly max out in their capacity to test for the new virus.

The newly developed NeuMoDx^TM^ device (NeuMoDx inc. Ann Arbor, USA, distributed by QIAGEN in Europe and other markets) is a fully automated, random access real-time PCR-based diagnostic platform that allows the use of “Laboratory Developed Tests” (LDT). The aim of this study was to evaluate the sensitivity, specificity and capacity of the *NeuMoDx N96* system for the qualitative detection of SARS-CoV-2 using a commercially available assay.

## Material and Methods

### Reference material and reagents

Positive material was in-vitro transcribed RNA containing the amplicon region of SARS-CoV-2 (GenExpress, Berlin, Germany), cell culture supernatants containing inactivated SARS-CoV-

2 provided by the Institute of Virology of the Charité Berlin as well as one clinical sample (respiratory specimen). UTM®, Universal Transport Medium™, (Copan Diagnostics Inc., Brescia, Italy) media (3 ml) was spiked with a predefined amount of artificial RNA. These specimens were used as starting material. Artificial RNA (commonly used as a positive control for PCR assays) was used for the initial evaluation of the *NeuMoDx N96 system*. As it was clear that artificial RNA does not reflect clinical material we additionally tested cell culture supernatant containing inactivated SARS-CoV-2 and a clinical sample. Dilutions from 10e^5^ to 10 copies per mL for artificial RNA targeting the E-Gene target and dilutions from 1.6e^2^ to 0.16 copies per µL of inactivated SARS-CoV-2 RNA were analyzed. In addition, serial dilution of a respiratory specimen were tested. Virus detection was performed on both systems using SARS-CoV (COVID19) E-gene kit 53–0776-96 (TIB Molbiol, Berlin, Germany). The assay is based on FAM (Fluorescein amidites) labeled hydrolysis probes which can detect a 76 bp long fragment from a conserved region in the E gene of SARS-CoV-2.

### Routine assay: Real-time PCR testing using the Roche LightCycler 480II

Samples analyzed with the *LightCycler* 480II system (Roche, Vienna, Austria) were extracted with a Roche MagNa Pure compact system (200 µL sample volume, 50 µL elution volume). The PCR was carried out according to the recently published protocol of Corman et al. [2]. Five µL of the extract was added to 20 µL PCR volume, which included the Roche LightCycler Multiplex RNA Virus Master (Roche Diagnostics, Switzerland). In addition, 0.5 µL of the EAV RNA Control Reaction was added (LightMix® Modular EAV RNA Extraction Control, Roche). Thermal cycling was performed at 55 °C for 10 min for reverse transcription, followed by 95 °C for 3 min and then 45 cycles of 95 °C for 15 s, 58 °C for 30 s.

### Real-time PCR testing using the NeuMoDx N96 molecular system

The *NeuMoDx N96* platform allows the application of laboratory-developed tests by using the so-called LDT strips for primer/probe addition. LDTs are diagnostic tests designed, manufactured and used by a single laboratory and are often implemented if no commercial test exists or when an existing test does not meet current clinical needs. The NeuMoDx LDT Primer/Probe Strip is an empty, foil-covered, disposable plastic strip with 16 wells into which the user pipets assay-specific primers and probe(s) to process LDTs on a NeuMoDx System. The NeuMoDx System represents a sample-to-result device which combines reagent storage, nucleic acid extraction, PCR setup, amplification, and detection, as well as results analysis and reporting. Reagents are dried and stable at room temperature. The NeuMoDx system incorporates a universal nucleic acid isolation chemistry based on magnetic beads enabling extraction of qPCR ready RNA from several specimens, combined with a sensitive quantitative rt-PCR assay. For our laboratory-developed protocol, 5 µL of the primer/probe mix (equally concentrated to the routine assay) was added to the LDT strip, which contains 16 positions for primer/probes. The device itself can be loaded with multiple LDT strips. Another strip, the so-called LDT RNA Master, contains the Mastermix as well as the primer/probes for the sample process control, which is performed as a duplex PCR reaction. For this study 200 µL of sample material was used for each PCR run. The thermal cycling profile was: Reverse Transcription was performed at 50°C for 15 min, followed by 95°C for 4 min and then 50 cycles of 95°C for 6 s, and 60°C for 19s.

## Ethical statement

The institutional ethics committee was informed. In the current situation, no formal ethic statement was required.

## Results

### NeuMoDx N96 system for PCR- based detection of SARS-CoV-2

The *NeuMoDx N96 system* detected all concentrations of the dilutions of the artificial RNA except for the lowest concentration which was detected only once. All of the concentrations of the dilutions of the cell culture supernatants containing inactivated SARS-CoV-2 and the clinical sample were detected. For the artificial RNA the 10e^5^ concentration (copies per mL) yielded a mean Ct value of 23.9, for 10e^4^ 27.4, for 10e^3^ 31.0, for 10e^2^ 34.0, and for 10 copies per mL a Ct-value of 41.6 was observed. For the cell culture supernatant containing inactivated SARS-CoV-2 the 1.6e^2^ concentration yielded Ct values of 24.1/25.8, for 1.6e^1^ 29.9/28.9, for 1.6e°^.1^ 32.1/32.6, and for 1.6e°^.01^ copies per µL Ct-values of 37.3/34.6 were observed (Figure 1). The undiluted clinical sample displayed a Ct-value of 23.4 in the *LightCycler 480II system*. For the respiratory specimen the 1:10 dilution yielded a Ct value of 22.8, for 1:100 24.6, for 1:1,000 29.3, for 1:10,000 34.8, and for 1:100,000 a Ct-value of 34.6 was observed with the *NeuMoDx N96* system. The *NeuMoDx N96* system displayed an equal or better detection rate for SARS-CoV-2 compared with the *LightCycler 480II* system (Table 1).

**Table 1.** Ct-Values of the *LightCycler480 system* and the *NeuMoDx N96* for the detection of in-vitro transcribed RNA containing the amplicon region of SARS-COV2 (three measurements for each sample), cell culture heat inactivated SARS-COV2 (two measurements for each sample) and respiratory specimen of a patient with confirmed COVID-19 (one measurement for each sample), according to a dilution row series. Every given Ct-value indicates one PCR run. Bottom section shows the assessment of the specificity. ^#^The 89 bp long artificial RNA fragment was too short for the extraction with the “MagNa Pure compact system”; ^§^The precision of Ct-values are attenuated at extremely low virus concentrations. *refers to the Ct-value of the *LightCycler 480II.*

RNA concentration	Ct-Values Lightcycler 480II	Ct-Values NeuMoDxN96
In-vitro transcribed RNA		
10e^5^ (cp/ml)	not performed	23.3/24.2/24.2
10e^4^ (cp/ml)	not performed	27.4/27.4/27.4
10e^3^ (cp/ml)	not performed	30.6/31.6/30.7
10e^2^ (cp/ml)	not performed	34.5/33.5/33.9
10e^1^ (cp/ml)	not performed	neg/41.6/neg
Inactivated cell culture SARS-COV2		
1.6e^2^ (cp/µl)	29.78/29.7	24.1/25.8
1.6e^1^ (cp/µl)	32.5/32.5	29.9/28.9
1.6e°^.1^ (cp/µl)	34.47/34.9	32.1/32.6
1.6e°^.01^ (cp/µl)	Neg/neg	37.3/34.6
Clinical sample (respiratory specimen, undiluted Ct 23.4*)		
1:10	25.5	22.8
1:100	28.8	24.6
1:1,000	32.7	29.3
1:10,000	36.8	34.8
1:100,000	neg	34.6
Assessment of Specificity		
Influenza A	neg	neg
Influenza B	neg	neg
RSV	neg	neg

If a test yielded an invalid test result (i.e. indeterminate or unresolved) the test was repeated from the same aliquot. Two out of the 28 PCR runs of the dilution had to be repeated on the *NeuMoDx* N96 *system* because of an indeterminate/unresolved PCR test result. None of the tests performed on the *LightCycler 480II system* had an invalid test result. For the assessment of the specificity of both test systems, we used positive sample material from international external quality assessment proficiency testing panels provided by QCMD (Quality Control for Molecular Diagnostics) for Influenza A, Influenza B and respiratory syncytial virus (RSV) and both test systems displayed a specificity of 100%.

### Time to result on the NeuMoDx N96 system

The median PCR run time for all 28 PCR runs was 91 (IQR 84–97) minutes. The inclusion of two repeated PCRs runs increased the median time to 98 (IQR 91–103) minutes. The handling time, which refers to the pipetting of the master-mix into each well of the strip, was approximately 3 minutes for one sample and 25–30 minutes for 32 samples.

## Discussion

Fast and sensitive detection of SARS-CoV-2 overcomes diagnostic uncertainty and leads to the rapid implementation of infection control measures and, if in the future available, to early administration of antiviral medication. Molecular testing of SARS-CoV-2 is however not only indispensable for providing healthcare but also crucial for understanding the trajectory and severity of Covid-19. Patients with asymptomatic infections with SARS-CoV-2 are more frequent than initially anticipated and these patients could easily accelerate the current epidemic situation [5] since asymptomatic patients show a similar viral load in respiratory specimens compared to symptomatic patients [6]. Therefore, a high transmission potential of asymptomatic patients or those with a mild/subclinical infection can be suspected, especially since these individuals are likely not to receive molecular testing for the presence of SARS-CoV-2. In addition, the transmission might often occur in the early phase of the infection, when the viral load of respiratory fluids is at its peak, but the patient still asymptomatic [6]. Taken together, these findings strongly support the standpoint, that, at least in areas with high infection rates, testing for SARS-CoV-2 is of high importance.

The evolution of (partially) automated testing techniques led to a significant improvement of patients’ care, by reducing the turnaround time from sample collection to definitive clinical diagnosis. Apart from reducing the turn-around time, a fully automated PCR further leads to an increase in sensitivity, reproducibility and efficiency for pathogen detection [7,8]. Sensitive testing for SARS-CoV-2 is highly important for triage, infection control measures, and infection

control strategies. Ideally, the test itself should be efficient and flexible for the usage in a clinical laboratory. Our approach is based on a common real-time PCR amplification workflow performed on the *NeuMoDx N96 system* using already validated primers and probes specific for the detection of SARS-CoV-2. The major advantages of the *NeuMoDx N96 system* are the short turnaround time, the high capacity as well as the possibility of random access and prioritization of samples (STAT) capabilities. STAT capabilities allow to prioritize emergency samples at any time, whereas random access enables processing up to 30 regulated assays simultaneously. In addition, the *NeuMoDx N96 system* allows the implementation of LDT assays, which is – given the current pandemic situation of COVID-19 – of special value to provide fast and high-throughput PCR diagnostics. Given that one test (sample-to-result) takes around 80 minutes, we estimate that the *NeuMoDx N96* – with a loading capacity of 96 samples per run – is capable of performing approximately 435 tests within 24 hours without being restricted to batch testing. In this study 2 out of 28 of the tests displayed an invalid result on the *NeuMoDxN96* system and had to be repeated to achieve valid results, but all of the repeated samples showed a final valid test result. Using the commercially available assay, the capacity of the *NeuMoDx N96* could easily surpass the capacity of most currently used molecular test systems and significantly reduce personnel engagement times and manual steps. An important limitation of our study is the limited number of clinical specimens tested with the *NeuMoDx N96* system. Future large-scale method comparison studies will be necessary to evaluate the stability and reproducibility of the SARS-CoV-2 detection with with the *NeuMoDx N96* system. Taken together, the results of our study show that the *NeuMoDx N96 system* is a compelling option for high-throughput PCR analysis for the detection of SARS-CoV-2.

## Supplementary Material

Supplemental Material

Supplemental Material
