# Supplementary material for: Development of a fully automated high throughput PCR for the detection of SARS-CoV-2: The need for speed
Source: Virulence. 2020 Jul 29;11(1):964–7. doi: 10.1080/21505594.2020.1798041 (PMC7549918; doi:10.1080/21505594.2020.1798041)
Supplement: Supplemental Material [file KVIR_A_1798041_SM0059.docx]

**Legend to Figure I (Supplemental Material)**

Observed PCR amplification curves from a single run of cell culture supernatant containing inactivated SARS-CoV-2. Numbers indicate the Ct-values observed for each dilution: 1= 1.6e^2^ copies per µL, Ct value: 25.8; 2=1.6e^1^ copies per µL, Ct value: 28.9; 3= 1.6e^0.1^ copies per µL, Ct value: 32.1; 4=1.6e^0.01^ copies per µL, Ct-value of 37.3; 5=negative control. Yellow lines indicate sample process control (SPC).
