# Supplementary figures and images for: Development of a fully automated high throughput PCR for the detection of SARS-CoV-2: The need for speed
Source: Virulence. 2020 Jul 29;11(1):964–7. doi: 10.1080/21505594.2020.1798041 (PMC7549918; doi:10.1080/21505594.2020.1798041)

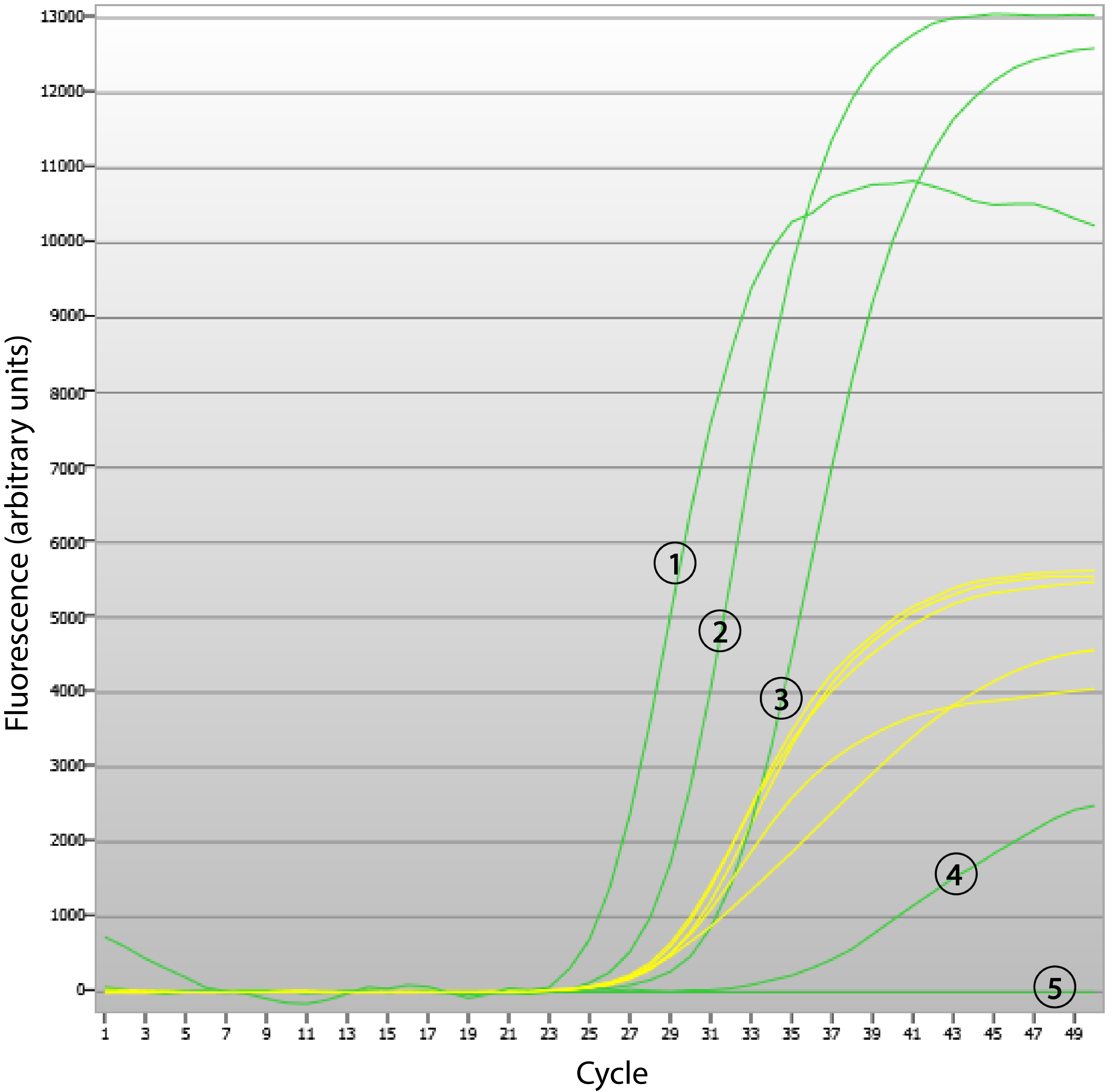

Supplement: Supplemental Material [file KVIR_A_1798041_SM0058.png]
